# Production and Characterization of High Value Prebiotics From Biorefinery-Relevant Feedstocks

**DOI:** 10.3389/fmicb.2021.675314

**Published:** 2021-04-29

**Authors:** Kalavathy Rajan, Doris H. D’Souza, Keonhee Kim, Joseph Moon Choi, Thomas Elder, Danielle Julie Carrier, Nicole Labbé

**Affiliations:** ^1^Center for Renewable Carbon, The University of Tennessee Institute of Agriculture, Knoxville, TN, United States; ^2^Department of Food Science, The University of Tennessee Institute of Agriculture, Knoxville, TN, United States; ^3^USDA-Forest Service, Southern Research Station, Auburn, AL, United States; ^4^Department of Biosystems Engineering and Soil Science, The University of Tennessee Institute of Agriculture, Knoxville, TN, United States; ^5^Department of Forestry, Wildlife and Fisheries, The University of Tennessee Institute of Agriculture, Knoxville, TN, United States

**Keywords:** Hemicellulosic oligosaccharides, hybrid poplar, switchgrass, southern pine, batch fermentation, *Lactobacillus casei*, *Bifidobacterium bifidum*, *Bacteroides fragilis*

## Abstract

Hemicellulose, a structural polysaccharide and often underutilized co-product stream of biorefineries, could be used to produce prebiotic ingredients with novel functionalities. Since hot water pre-extraction is a cost-effective strategy for integrated biorefineries to partially fractionate hemicellulose and improve feedstock quality and performance for downstream operations, the approach was applied to process switchgrass (SG), hybrid poplar (HP), and southern pine (SP) biomass at 160°C for 60 min. As a result, different hemicellulose-rich fractions were generated and the chemical characterization studies showed that they were composed of 76–91% of glucan, xylan, galactan, arabinan, and mannan oligosaccharides. The hot water extracts also contained minor concentrations of monomeric sugars (≤18%), phenolic components (≤1%), and other degradation products (≤3%), but were tested for probiotic activity without any purification. When subjected to batch fermentations by individual cultures of *Lactobacillus casei*, *Bifidobacterium bifidum*, and *Bacteroides fragilis*, the hemicellulosic hydrolysates elicited varied responses. SG hydrolysates induced the highest cell count in *L. casei* at 8.6 log_10_ cells/ml, whereas the highest cell counts for *B. fragilis* and *B. bifidum* were obtained with southern pine (5.8 log_10_ cells/ml) and HP hydrolysates (6.4 log_10_ cells/ml), respectively. The observed differences were attributed to the preferential consumption of mannooligosaccharides in SP hydrolysates by *B. fragilis*. *Lactobacillus casei* preferentially consumed xylooligosaccharides in the switchgrass and southern pine hydrolysates, whereas *B. bifidum* consumed galactose in the hybrid poplar hydrolysates. Thus, this study (1) reveals the potential to produce prebiotic ingredients from biorefinery-relevant lignocellulosic biomass, and (2) demonstrates how the chemical composition of hemicellulose-derived sources could regulate the viability and selective proliferation of probiotic microorganisms.

## Introduction

Hemicellulose, a structural polysaccharide constituting 9–34% of lignocellulosic biomass (Yadav et al., 2018), is often an undervalued and underutilized stream of biorefinery processes with untapped potential in platform chemicals, and food and cosmetic industries (Takkellapati et al., 2018; Lolou and Panayiotidis, 2019). The native structure of hemicellulose varies depending on the plant species, with the predominant forms in herbaceous, hardwood, and softwood biomass being arabinomethylglucuronoxylan, methylglucuronoxylan, and galactoglucomannan, respectively (Spiridon and Popa, 2008). Their unique structure and chemical composition create new opportunities for diversification and high-value nutraceutical applications such as prebiotics.

Prebiotics are indigestible oligosaccharides commonly produced from food sources such as chicory root, milk, and oats (Davani-Davari et al., 2019), and are known to promote the growth of beneficial probiotic microorganisms in the lower intestine of mammals. Hemicellulosic oligosaccharides (HOS) prepared from lignocellulosic biomass have also been reported to impart similar benefits. HOS, composed of galactoglucomannan and arabinoglucuronoxylan isolated from Norway spruce and birch wood, were reported to selectively induce the proliferation of *Bifidobacteria* and in turn improve the production of short chain fatty acids like butyric and propionic acids (La Rosa et al., 2019). Similarly, xylooligosaccharides produced from *Miscanthus* were reported to sustain the growth of *Lactobacillus brevis*, as well as promote the production of lactic and acetic acids (Hong et al., 2019). *In vitro* and *in vivo* studies have shown that supplementation with xylooligosaccharides could provide additional benefits, including reduction of inflammatory cell signaling pathways (Hansen et al., 2013) and improvement in gut barrier functions (Thiennimitr et al., 2018). Together, these physiological changes have been reported to mitigate obesity (Thiennimitr et al., 2018), colon cancer (Hsu et al., 2004), type-2 diabetes (Yang et al., 2015) and improve the overall immune response (Pham et al., 2018). Hence, investigating the prebiotic potential of HOS derived from renewable lignocellulosic feedstocks would pave way for achieving new health benefits as well as generate new revenue streams for biorefineries. Moreover, the demand for prebiotic ingredients is expected to reach 1.35 million tons by 2024 (Ahuja and Deb, 2017), therefore, complementing food sources with lignocellulosic feedstocks would make the prebiotic ingredient industry more sustainable.

Different biorefineries will utilize different regionally available lignocellulosic feedstocks, and for a sustainable year-round operation it may even be essential to switch between feedstocks (Baral et al., 2019). Hence, it is necessary to investigate the efficacy of prebiotics production from multiple sources. Hybrid poplar (HP) and switchgrass (SG) are dedicated energy crops, with field trials in the United States averaging a productivity of 15 ton ha^−1^ yr^−1^ (Volk et al., 2018) and 10 ton ha^−1^ yr^−1^ (Kim et al., 2020), respectively. These crops grow rapidly on a range of sites including marginal land and former industrial sites, providing numerous environmental benefits such as low carbon footprint, enhanced water and soil quality, as well as creating diverse landscapes that support biodiversity (Townsend et al., 2018). Complementing energy crops with regional feedstocks, such as southern pine (SP) whose net production in 2015 was 132 million wet tons in the Southern United States (Gray et al., 2018), would enhance the development of sustainable supply chains for various bioconversion platforms (Edmunds et al., 2018). Hence, these three biorefinery-relevant feedstocks were chosen for this study and to our knowledge, this is the first time they have been investigated for prebiotic production potential.

Of the different strategies employed to fractionate hemicellulose from lignocellulosic biomass, including alkaline (Geng et al., 2018), dilute acid (Rusanen et al., 2019), and hot water hydrolysis (Gallina et al., 2018), the use of hot water has dual advantages of improving the feedstock quality and performance for downstream conversion (Wells et al., 2020) and also requiring no chemical inputs (Kim et al., 2009). Hot water pretreatment reduces inorganic impurities and improves biomass combustion quality (Liu et al., 2018), as well as reduces biomass recalcitrance and enhances fermentable sugar production (Wells et al., 2020). Moreover, up to 95% of hemicellulose could be fractionated from lignocellulosic biomass using hot water extraction (HWE) at 160–170°C, for 40–120 min (Krogell et al., 2013; Gallina et al., 2018). The liquid hydrolysates fractionated during HWE are enriched in HOS and could either be used directly or after partial purification for prebiotic applications (Chen et al., 2015). Since the source of HOS has been shown to have a significant effect on ensuing probiotic activity (Hong et al., 2019; La Rosa et al., 2019), it is essential to understand how the structure and compostion of HOS derived from different biorefinery-relevant feedstocks will impact both the quality and level of prebiotic production.

In this study, we conducted extensive characterization of HOS fractionated from SG, HP, and SP, and investigated their capability to sustain individual cultures of probiotic microorganisms. Probiotic bacteria belonging to *Lactobacillus* and *Bifidobacterium* genera are naturally found in the small and large intestine of humans (Ng et al., 2014), and hence, *Lactobacillus casei* and *Bifidobacterium bifidum* were selected as our test systems. *Lactobacillus casei* has been proven to proliferate in dairy products, xylo-oligosaccharides and fructo-oligosaccharides (Hill et al., 2018), whereas *B. bifidum*, a key member of infant gut microbiota (~10%), is known to grow in the mucosal barrier (Turroni et al., 2020) and to establish well in galacto- and fructo-oligosaccharides (Sims and Tannock, 2020). Both these bacteria have never been tested for their ability to ferment switchgrass, hybrid poplar, or southern pine-derived HOS. In addition to using these well-established microorganisms, an emerging probiotic bacterium *Bacteroides fragilis*, which has exhibited the potential to utilize xylooligosaccharides, was evaluated in our study (La Rosa et al., 2019). The phylum *Bacteroidetes* have been reported to dominate the adult human gut by 26–32% and also aid in the fermentation of complex carbohydrates, proteins, and fats (Poeker et al., 2018), thereby promoting overall well-being. Hence, by investigating the efficacy of HOS fermentation by these selected probiotic bacteria, we could provide insights about how the structure and composition of HOS, isolated from different biorefinery-relevant lignocellulosic feedstocks, could affect their *in vitro* proliferation. Our long term goal is to establish HOS structure-function relationship and thereby, optimize the HWE of high-quality prebiotics from lignocellulosic feedstocks.

## Materials and Methods

### Biomass

Pulp grade chips of debarked hybrid poplar (*Populus deltoides* × *Populus trichocarpa*) and southern pine (*Pinus taeda* L.) wood were obtained from the Center for Renewable Carbon (Knoxville, TN), and Auburn University (Auburn, AL), respectively. The average size of wood chips was 4 cm^2^ and 0.5 cm in thickness. Chopped stalks and leaves of switchgrass (*Panicum virgatum* L.) Alamo variety were obtained from Genera Energy LLC (Vonore, TN). The average particle size of the switchgrass biomass was 0.48 cm. All biomass materials were reduced to a particle size of 0.43 mm (40-mesh) using a Thomas Wiley® mini-blade mill (Swedesboro, NJ) for chemical characterization purposes. The chemical composition of starting biomass materials is provided in Table 1.

**Table 1:** Chemical composition of lignocellulosic biomass on extractives-free basis.

Feedstock	Biomass composition (% oven dry wt. basis)^*^						
	Cellulose	Hemicellulose	Acid insoluble lignin	Acid soluble lignin^#^	Acetyl	Ash	Mass closure
Switchgrass (SG)	40.0 ± 1.6	28.9 ± 1.8	15.3 ± 0.3	7.7 ± 0.4	4.8 ± 0.2	2.6 ± 0.1	99.3 ± 2.6
Hybrid poplar (HP)	46.3 ± 0.5	23.6 ± 0.5	19.4 ± 0.5	6.4 ± 0.1	3.6 ± 0.2	1.1 ± 0.0	100.4 ± 0.6
Southern pine (SP)	38.6 ± 0.2	21.9 ± 0.1	32.7 ± 0.4	2.5 ± 0.1	2.9 ± 0.1	0.5 ± 0.0	99.1 ± 0.9

* Mean and SD provided for *N* = 3. Chemical composition was determined using National Renewable Energy Laboratory’s (Golden, CO) standard protocols (Sluiter et al., 2010).

# Acid soluble lignin content of SG was measured at 320 nm, whereas for SP and HP at 240 nm.

### Hot Water Extraction of Hemicellulosic Sugars

**Figure 1:**
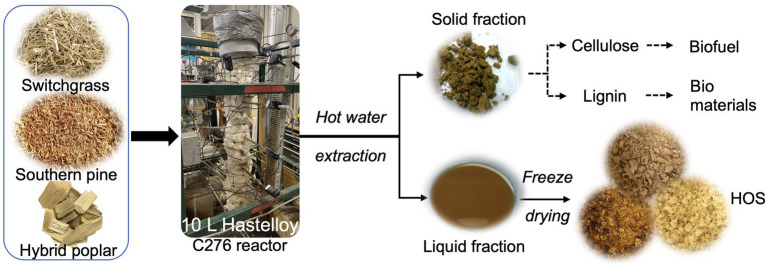
Production scheme of hemicellulosic oligosaccharides (HOS) from switchgrass (SG), hybrid poplar (HP), and southern pine (SP) biomass, using hot water extraction (HWE) at 160°C for 60 min. HOS is a co-product of biomass fractionation, and development of value-added prebiotic ingredients is a strategy for minimizing waste and increasing revenue in a biorefinery.

Prior to hot water extraction, all biomass materials were pre-conditioned by first heating with water and then with 95% ethanol, at 100°C for 60 min each, to remove any extractives (inorganic and organic non-structural components). Afterward, 750 g of the conditioned biomass materials were added to an in-house constructed 10 L Hastelloy C276 pressure reactor (Bozell et al., 2011) and loaded with 5.5 kg of water, such that the average solid loading was 14% w/w. HWE was then conducted at 160°C for 60 min, where the treatment duration was counted after the reactor reached the required temperature. The parameters for HWE were chosen based on our previous work (Wang et al., 2017). Post HWE, the reactor was cooled to 30°C and drained; all liquid hydrolysates were collected, filtered to remove any particulates, and then freeze-dried under reduced pressure (0.2 mbar) at −44°C (Labconco FreeZone 4.5 L system, Kansas City, MO). The freeze-dried product thus obtained was termed hemicellulosic oligosaccharides or “HOS.” A schematic representation of HOS production is given in Figure 1. The HOS yield was calculated as follows:

(1) $$HOS\;yield\;\left(\%\right)=\frac{Mass_{freezedriedsolids}\;\left(g\right)}{Mass_{originalbiomass}\;\left(g\right)}\times 100$$

#### Chemical Characterization of HOS

The monosaccharide composition of HOS was determined using a high-performance liquid chromatography (HPLC) system fitted with an Aminex HPX-87P analytical column (Bio-Rad Laboratories Inc., Hercules, CA) and a refractive index (RI) detector (Flexar, PerkinElmer, Waltham, MA). The column was maintained at 85°C with an eluent flow rate of rate of 0.25 ml/min. The system was calibrated using commercial sugar standards for xylose (Xyl), glucose (Glc), mannose (Man), arabinose, and galactose. The byproducts composition was measured using the same HPLC system (Flexar, PerkinElmer, Waltham, MA), but fitted with an Aminex HPX-87H analytical column and a photo diode array detector, calibrated using acetic acid, furfural, 5-hydroxymethylfurfural and formic acid standards purchased from Alfa-Aesar (Haverhill, MA).

The total oligosaccharide concentration was determined after digestion of the HOS with 4% (w/w) sulfuric acid solution at 121°C for 1 h; this process depolymerizes the oligosaccharides into monomeric sugars, which are then quantified using the same monosaccharide-detecting HPLC-RI method (Sluiter et al., 2008).

The total phenolic content of our HOS preparations was determined using the Folin-Ciocalteu method (Rajan and Carrier, 2016), where 200 μl of the 0.2 N phenol reagent (MP Biomedicals, Irvine, CA) was added to 100 μl of 1.25 g/L HOS and incubated in the dark for 5 min. Afterward, 700 μl of 7.5% sodium carbonate solution (Alfa-Aesar, Haverhill, MA) was added to the mixture and incubated in the dark at room temperature for 2 h. For the instrument calibration, 100 μl of gallic acid solution in 95% methanol (Spectrum™, Gardena, CA) was used, at a concentration range of 0.04–0.2 g/L. A blank containing deionized water was also included. After 2 h of incubation, absorbances of the blank, calibration standards, as well as the HOS preparations were recorded at 765 nm using UV-Vis spectrophotometer (Lambda 650, PerkinElmer, Duluth, GA). The total phenolic content of HOS was expressed as gallic acid equivalent (i.e., g GAE/L).

### Culturing Probiotic Bacterial Stocks

*Bifidobacterium bifidum* (ATCC® 29521™) and *Bacteroides fragilis* (ATCC® 25285™), purchased from the American Type Culture Collection (Manassas, VA), were cultured in modified reinforced clostridial medium (ATCC® Medium #2107) and modified chopped meat medium (ATCC® Medium #1490), respectively. These bacteria were incubated in a static air-tight chamber, at 37°C, that utilizes a GasPak™ EZ pouch system (Becton, Dickinson & Co., Sparks, MD) to generate anaerobic conditions. *Lactobacillus casei* was obtained from the culture collection of the Department of Food Science, the University of Tennessee Institute of Agriculture (Knoxville, TN), and grown in tryptic soy broth (TSB) medium at 37°C under aerobic conditions. The TSB medium was prepared using casein peptone (17 g/L), sodium chloride (5 g/L), soy peptone (3 g/L), dextrose (2.5 g/L), and dipotassium phosphate (2.5 g/L) purchased from Alfa-Aesar (Haverhill, MA), where the pH was adjusted to 7.1. Isolated colonies were obtained and maintained on respective media.

### Prebiotic Activity Assays

The *in vitro* fermentation assays were conducted using M9 minimal salts medium (Sigma-Aldrich, St. Louis, MO), which was prepared with 4 g/L of carbon source, 2 mM MgSO_4_, 0.1 mM CaCl_2_, and adjusted to pH 7.1. Glucose, Xyl, or Man were used as controls for the carbon source, whereas the treatment groups contained 4 g/L of switchgrass, hybrid poplar, or southern pine HOS. All media preparations were cold sterilized by passing through a 0.2 mm filter membrane under reduced pressure. Fresh stock cultures of all three probiotic bacteria having an average count of 1 × 10^11^ cells/ml were used as inoculum. *Lactobacillus casei* and *B. fragilis* were cultured in a high throughput assay, where 10 μl of inocula were added to 200 μl of modified M9 media in a 96-microwell plate and then incubated in a microplate reader (Synergy H1, BioTek Instruments, Burlington, VT) at 37°C for 120 h. Bacterial growth was measured as optical density (OD) by the microplate reader every 30 min at 600 nm. There were three biological replicates per bacterium per carbon source and each biological replicate had two technical replicates, totaling six replicates. A media blank was also included and OD_600_ readings taken at 0 h were used as the baseline to subtract from further measurements.

In the case of *B. bifidum*, which is not compatible for the high-throughput assay, 5 ml media preparations taken in N_2_-flushed 10 ml glass tubes were used. After adding 250 μl of the prepared inoculum, these tubes were incubated at 37°C for 120 h. Samples were manually collected every few hours and the OD_600_ was measured using the Synergy H1 microplate reader. These experiments were also repeated six times. Anaerobic conditions were induced for both *B. fragilis* and *B. bifidum* by sealing BD GasPak™ EZ pouches inside the 96-well plates or the air-tight growth chambers. The OD_600_ readings were calibrated with bacterial cell counts obtained from a BD FACSAria III flow cytometer (Sparks, MD) fitted with a Sapphire 488-50 blue laser system (see Supplementary Figure S1). The prebiotic activity was generally expressed as total cell counts per ml of media. Batch fermentations on a 10 ml scale were also performed in order to collect spent media every 24 h for further analyses.

#### Chemical Characterization of Fermentation Media

The composition of specific oligosaccharides contained in the HOS preparations (modified minimal salts media) was determined using a Dionex™ ICS-6000 high-performance anion exchange chromatography (HPAEC) system (Thermo Fisher Scientific, Madison, WI) fitted with a CarboPac™ PA200 analytical column (250 mm × 3 mm) and a corresponding microbore guard column (50 mm × 3 mm). The pulsed amperometric detection (PAD) system of ICS-6000 had a AgCl reference electrode and a gold working electrode. The mobile phases were composed of solvent A: 100 mM NaOH, and solvent B: 100 mM NaOH mixed with 320 mM sodium acetate. A gradient elution method was used as follows; hold 100% solvent A for 15 min, then ramp to 50% solvent B at a linear rate for 40 min, afterward increase solvent B to 100% in 1 min and hold constant for 4 min; finally, return the mobile phase to 100% solvent A in 1 min. The eluent flow rate was 0.5 ml/min, injection volume was 10 μl, and the column temperature was 35°C. Pure standards (>95%) of cellobiose, xylobiose, xylotriose, xylotetraose, xylopentaose, xylohexaose, mannobiose, mannotriose, arabinobiose, and arabinotriose, purchased from Megazyme (Wicklow, Ireland), were used to calibrate the instrument.

## Results and Discussion

### Fractionation and Characterization of HOS

Our previous work has shown that HWE procedure at 160°C for 60 min could be utilized to pretreat lignocellulosic biomass, namely hybrid poplar, switchgrass, and pine bark (Wang et al., 2017; Liu et al., 2018), to decrease inorganic impurities and reduce recalcitrance such that the overall biomass quality was enhanced for subsequent thermo- and biochemical conversion processes. The liquid hydrolysate obtained as a co-product during HWE is enriched in HOS, which could either be utilized for ethanol fermentation after extensive treatments (Phaiboonsilpa et al., 2020) or directly used for high-value applications such as prebiotics. In this work, we determined that HWE of SG, HP, and SP biomass produced 11.9 ± 0.2, 10.8 ± 0.2, and 12.3 ± 0.1% of HOS yields (Equation 1), respectively. The corresponding hemicellulose extraction efficiencies for SG, HP, and SP were 41.2 ± 0.6, 45.8 ± 0.5, and 56.2 ± 0.2%, respectively, based on the original biomass composition (Table 1). These efficiencies are similar to previously reported pilot scale hemicellulose extraction, at ~50%, from hardwood and softwood biomass using hot water (Kilpeläinen et al., 2014).

The monosaccharide and total oligosaccharide compositions of SG, HP, and SP-HOS are presented in Table 2. The predominant oligosaccharide in SG and HP-HOS was xylan, while in SP-HOS it was mannan, which is similar to other HOS preparations from herbaceous, softwood, and hardwood species. HOS isolated from Norway spruce, a softwood species with galactoglucomannan backbone, was composed of 53% mannan and that from Norway birch, a hardwood species with arabinoglucuronoxylan backbone, was composed of 82% xylan (La Rosa et al., 2019). In the case of herbaceous species, such as *Miscanthus*, the hot water hydrolysates were reported to contain 63% xylan (Chen et al., 2015). Depending on their anatomical and chemical features, some feedstocks are more susceptible to hot water hydrolysis and hence, the corresponding HOS could depolymerize at an accelerated rate to form monosaccharides. The susceptibility of hemicellulosic backbone to autohydrolysis has been described as a function of degree of acetylation and the ratio of xylan to acid-insoluble lignin content (Lira, 2018). Accordingly, SG, which had comparatively higher number of acetyl groups as well as lower lignin content than HP (Table 1), produced higher amounts of monosaccharides during HWE. However, this was not the case when producing HOS from SP, which contained the highest concentration of monosaccharides at 184 g/L, despite the lower acetyl (2.9%) and higher acid-insoluble lignin content (32.7%) of SP biomass, indicating that further investigation is needed about glucomannan autohydrolysis kinetics.

**Table 2:** Total saccharide composition of hemicellulosic hydrolysates fractionated from SG, HP, and SP biomass using hot water.

Components (g/L)^*^	SG	HP	SP
Monosaccharides (DP = 1)			
Glucose Xylose Galactose Arabinose Mannose	3 ± 0 36 ± 1 - 47 ± 5 -	- 12 ± 0 34 ± 2 11 ± 0 -	41 ± 1 49 ± 2 25 ± 0 27 ± 0 42 ± 2
Oligosaccharides (DP ≤ 6)			
Cellobiose Xylobiose Xylotriose Xylotetraose Xylopentaose Xylohexaose Mannobiose Mannotriose Arabinobiose Arabinotriose	- 21 ± 1 14 ± 1 10 ± 0 4 ± 0 5 ± 0 - - 44 ± 2 18 ± 1	40 ± 2 13 ± 0 3 ± 0 3 ± 0 - - - - 21 ± 1 -	26 ± 1 38 ± 2 20 ± 0 14 ± 1 - - 28 ± 1 16 ± 0 25 ± 2 -
Oligosaccharides (DP > 6)			
^#^ Glucan Xylan Galactan Arabinan Mannan	56 ± 3 598 ± 16 74 ± 6 36 ± 3 6 ± 0	- 664 ± 14 83 ± 6 1 ± 0 77 ± 2	94 ± 2 32 ± 2 106 ± 3 10 ± 0 349 ± 15

* Mean and SD for *N* = 3; DP, degree of polymerization.

# Quantified indirectly upon hydrolysis by 4% sulfuric acid (Sluiter et al., 2008).

The HOS from SG, HP, and SP also contained degradation products of monosaccharides, formed under the high temperature and low pH conditions of HWE (Yan et al., 2016), namely acetic acid, formic acid, furfural, and 5-hydroxymethylfurfural. HP-HOS contained the lowest concentration of degradation products (Supplementary Table S1), whereas SP-HOS contained the highest concentration at 32 g/L, with formic acid (13 ± 1 g/L) and acetic acid (10 ± 1 g/L) sharing the majority. The total phenolic content, measured using the Folin-Ciocalteu method and expressed in gallic acid equivalent (GAE), was 10.3 ± 1.0, 7.4 ± 0.1, and 4.0 ± 0.1 g GAE/L for SG, HP, and SP-HOS, respectively. Presence of phenolic compounds indicated that lignin was also partially depolymerized from the biomass materials during HWE (Yan et al., 2016). Despite the presence of carbohydrate and lignin degradation products, we tested the HOS directly without any purification. This approach could decrease costs as well as preserve the HOS yield rates.

### Selective Proliferation of Probiotic Bacteria in HOS

We conducted *in vitro* fermentation studies with individual bacterial cultures of *L. casei*, *B. bifidum*, and *B. fragilis* in order to test the prebiotic potential of our SG-, HP-, and SP-HOS preparations. Xylose, glucose, and mannose sugars were used as control carbon sources in these tests. Previously, probiotic bacteria belonging to the genera of *Lactobacillus* and *Bifidobacterium* were successfully shown to proliferate in organosolv-pretreated cellulosic hydrolysates of birch and spruce wood (Karnaouri et al., 2019). Similarly, *in vitro* cultures of *L. brevis*, *B. adolescentis*, and *B. catenulatum* were reported to grow effectively in xylooligosaccharides fractionated using hot water from *Miscanthus* biomass (Chen et al., 2016; Hong et al., 2019). Moreover, *in vivo* studies have shown that bacteria belonging to *Bacteroidaceae* and *Prevotellaceae* could also assimilate xylooligosaccharides (Poeker et al., 2018). In our study, different probiotic bacteria grew at different rates in the HOS preparations.

**Figure 2:**
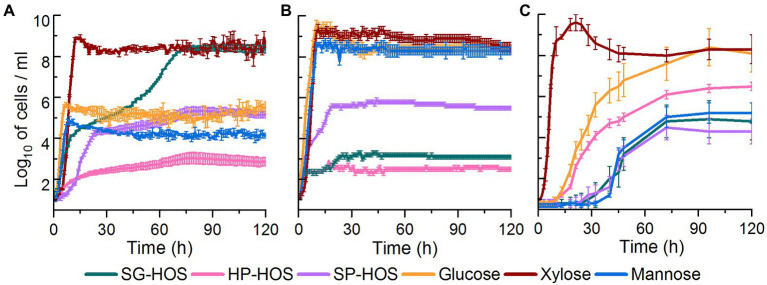
Growth curves of **(A)** *Lactobacillus casei*, **(B)** *Bacteroides fragilis*, and **(C)** *Bifidobacterium bifidum* cultivated in media containing 4 g/L of HOS isolated from SG, HP, and SP. Pure glucose (Glc), xylose (Xyl), and mannose (Man) solutions at 4 g/L were used as controls. Average cell counts and SDs are provided for *N* = 6. Growth curves were constructed after subtracting the cell counts in the inoculum.

As shown in Figure 2A, *L. casei* proliferated in all of the tested carbon sources, however, it displayed the highest cell counts in xylose and SG-HOS. *Lactobacillus casei*, being a facultative heterofermentative bacterium, has been previously reported to utilize a variety of carbon sources including xylose *via* the production of xylose isomerase and xylulose kinase enzymes (Chaillou et al., 1999). Specifically, strains with mutations affecting the phosphoenolpyruvate:mannose phosphotransferase system, have been reported to transport xylose *via* facilitated diffusion mechanism (Chaillou et al., 1999). *Lactobacillus casei* strains have also been reported to achieve a growth of ≤9 log_10_ cells/ml in enzymatic hydrolysates of coffee peel (Ratnadewi et al., 2020), Napier grass and other herbaceous biomass (Patipong et al., 2019) that were enriched in xylooligosaccharides (degree of polymerization, DP = 2–5). Therefore, the observed higher growth rates of *L. casei* could be attributed to its evolved tolerance in consuming xylose and xylooligosaccharides. Further investigation of *L. casei*’s sugar consumption profiles is presented in the ensuing sections.

*Bacteroides fragilis* achieved higher growth rates in the controls, as well as in SP-HOS, when compared to the other hot water hydrolysates (Figure 2B). *Bacteroides* species have been reported to preferentially utilize pentoses at high dilutions and under carbon-limited conditions (Degnan and Macfarlane, 1995). It has been also shown that substrate specific transport systems can be readily induced in the *Bacteroides* species. *Bacteroides fragilis*, specifically, has been reported to attain a cell density (OD_600_) of 0.6 in autohydrolysates of spruce wood that were enriched in acetylated galactoglucomannan (La Rosa et al., 2019). In our study, *B. fragilis* attained a concentration of 5.8, 3.2, and 2.9 log_10_ cells/ml, corresponding to OD_600_ of 0.32, 0.25, and 0.21, in media containing SP-, SG-, and HP-HOS, respectively.

*Bifidobacterium bifidum*, on the other hand, exhibited significant growth in xylose and glucose controls, as well as in HP-HOS, when compared to other substrates (Figure 2C). Previous investigations have shown that carbon resource utilization by *B. bifidum* is strain dependent and most strains can utilize glucose, lactose, galactose, mannitol, and xylose, *via* inducible polyol dehydrogenase and fructokinase enzymes, and convert them into lactate and acetate (de Vries and Stouthamer, 1968). Other *Bifidobacterium* species have also been reported to utilize xylooligosaccharides (DP = 3, 4) isolated from the autohydrolysates of corn cob, achieving a OD_600_ of 0.7 (Moura et al., 2007). In our study, *B. bifidum* attained a growth rate of 6.4, 4.8, and 4.3 log_10_ cells/ml, corresponding to OD_600_ of 0.37, 0.30, and 0.28, in fermentation media containing HP-, SG-, and SP-HOS, respectively. Elucidation of fermentation media components utilized by *B. bifidum* would provide further insights about how this bacterium persevered in the test systems.

Overall, it is evident that our crude HOS preparations could support the growth of various probiotic bacteria even when containing carbohydrates and lignin degradation products. SG-HOS induced diauxic growth in *L. casei*, thereby maximizing carbon resource utilization and achieving cell populations similar to that of the xylose control. It is important to note that SG-HOS contained the highest concentration of phenolic compounds at 10.3 g GAE/L. SP-HOS induced moderate growth of 4–6 log_10_ cells/ml in all three probiotic bacteria, despite containing the highest concentration of carbohydrate degradation products at 32 g/L (Supplementary Table S1). HP-HOS induced the highest growth in *B. bifidum*, but relatively underperformed with other bacteria even though it contained the least concentration of degradation products (Supplementary Table S1). Thus, no trend was observed between the concentration of carbohydrate and lignin degradation products and that of the bacterial growth rates. Previous studies have shown that the presence of phenolic compounds like vanillic acid, gallic acid, flavonoids, and catechins, at 1–7 g/L, did not influence the viability of *Lactobacillus* spp. and *B. bifidum* (Gwiazdowska et al., 2015; Pacheco-Ordaz et al., 2017). Similarly, the presence of acetic acid (5–19 g/L), formic acid (5 g/L), furfural (1–4 g/L), and 5-hydroxymethylfurfural (1–5 g/L), was shown to actually enhance the substrate utilization and metabolite production in certain heterofermentative lactic acid bacteria like *L. casei* (Gubelt et al., 2020; Abdel-Rahman et al., 2021; Giacon et al., 2021). Hence, the observed differences in growth rates may be attributed to the differential assimilation of HOS sugars, instead of inhibition by the degradation products, and a detailed investigation of the spent media will reveal which of the available xylo-, manno-, gluco-, and arabinooligosaccharides in the SG-, HP-, and SP-HOS were utilized by *L. casei*, *B. fragilis*, and *B. bifidum*.

### Properties of HOS Affecting Probiotic Bacterial Growth

#### Effect of Monosaccharide Composition

**Figure 3:**
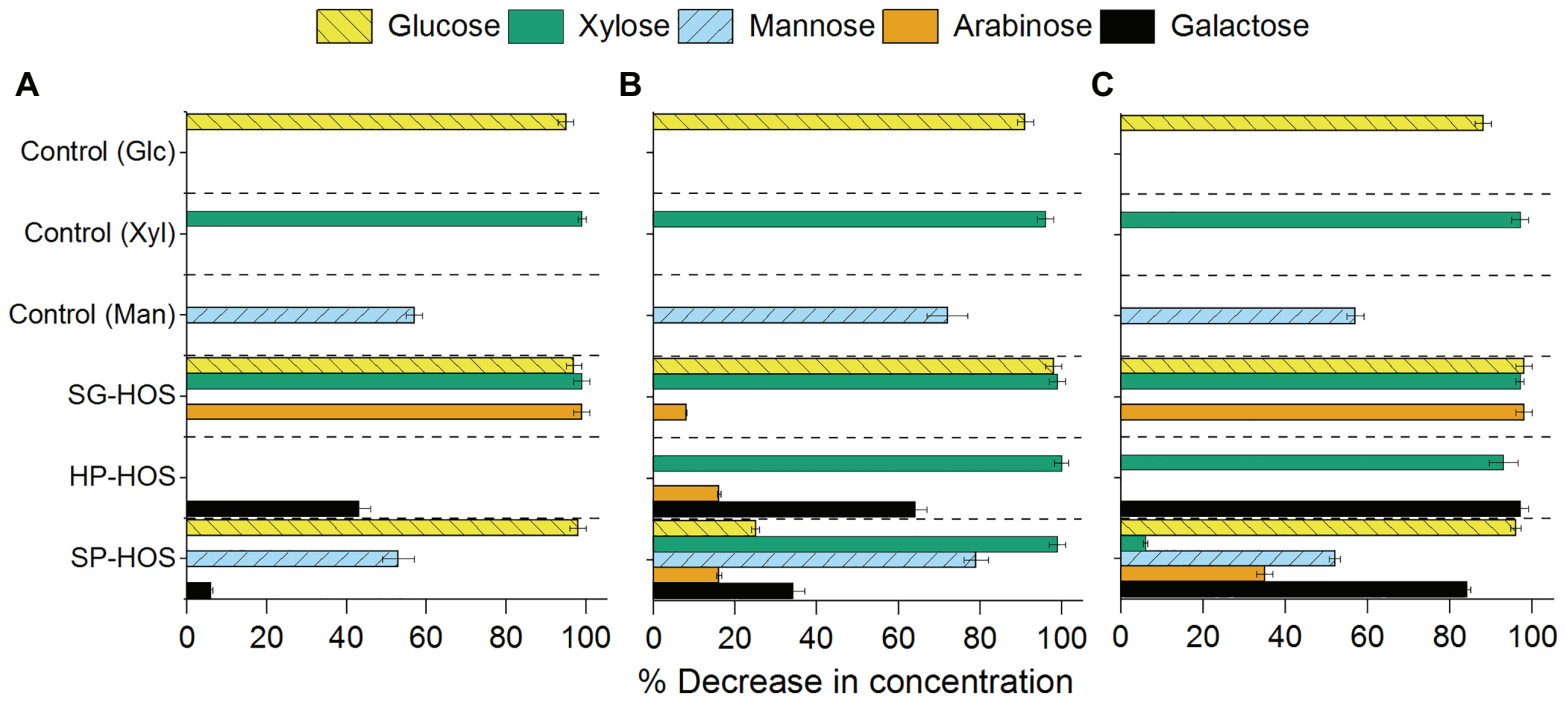
Monosaccharides consumed (in spent media) after 120 h of fermentation by **(A)** *L. casei*, **(B)** *B. fragilis*, and **(C)** *B. bifidum*. Average monomeric sugar utilization in the SG, HP, and SP HOS was determined by high-performance liquid chromatography (HPLC). Glucose, Xyl, and Man sugars were used as control carbon sources in the fermentation media. Error bars represent standard deviations for *N* = 3.

**Figure 4:**
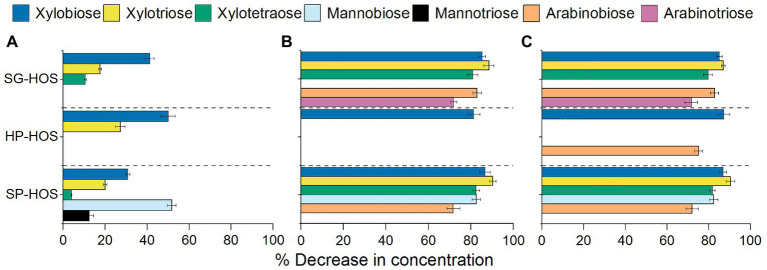
Characterization of oligosaccharides in the spent media after 120 h of fermentation by **(A)** *L. casei*, **(B)** *B. fragilis*, and **(C)** *B. bifidum*. Average sugar composition of media containing SG, HP, and SP HOS was determined using high-performance anion exchange chromatography (HPAEC)-pulsed amperometric detection (PAD) analysis. Error bars represent SDs for *N* = 3.

The analyses of the spent media after 120 h of fermentation by *L. casei*, *B. fragilis*, and *B. bifidum* by HPLC and HPAEC demonstrated that both mono- and oligo-saccharides in the HOS were utilized at different levels (Figures 3, 4). *Lactobacillus casei* utilized glucose and xylose to the fullest extent, both in the control as well as in our HOS preparations, but not mannose, which was consumed by only about 57% (Figure 3A). The same manno-phosphotransferase transport system favoring xylose diffusion in *L. casei* should also enable mannose uptake; however, previous studies have reported similar discrepancies between xylose and mannose uptake (Das et al., 2019). It was revealed that, despite improved transport, mannose metabolism was affected due to the downregulation of specific (LSEI_0681) genes under nutrient restricted conditions (Licandro-Seraut et al., 2014). Hence, the nutrient restricted conditions of the minimal salt media must have affected the mannose metabolic pathway in *L. casei* in an adverse manner. In the case of SG-HOS, where glucose, xylose, and arabinose consumptions were upregulated, *L. casei* was able to establish sufficient growth during the first log phase as shown in Figure 2A. The chemical analysis of the spent media, as a function of time, also showed that all monomers in SG-HOS were consumed within 8–24 h (Supplementary Figure S2).

*Bacteroides fragilis* exhibited improved mannose consumption by 18 and 44% in the control when compared to *L. casei* and *B. bifidum*, respectively. It also exhibited improved co-consumption of mannose, xylose, and galactose sugars in HP- and SP-HOS (Figure 3B). Previous studies have shown that *Bacteroides* species can co-assimilate xylose, glucose, galactose and arabinose in the presence of mannose (Degnan and Macfarlane, 1995). Hence, SP-HOS, which contained the highest concentrations of mannose and other monosaccharides, elicited the fastest growth rate in *B. fragilis*.

*Bifidobacterium bifidum* exhibited a longer lag phase than other probiotic bacteria, even in the glucose control, possibly due to the low concentration of carbon sources at only 4 g/L (Figure 2C). Although this bacterium originally evolved to metabolize galactooligosaccharides, previous studies have found genes encoding transporter and metabolic systems in different *B. bifidum* strains that enabled glucose, xylose and fructose utilization (Turroni et al., 2012). In our study, HP-HOS, which had the highest galactose concentration, promoted early *B. bifidum* establishment and reduced the lag time when compared to SG- or SP-HOS (Figure 2C). Even though this strain utilized different monosaccharides, including xylose, mannose and arabinose in different hydrolysates (Figure 3C), presence of galactose seemed to be crucial for achieving higher cell counts.

#### Effect of Oligosaccharide Composition

The tested probiotic bacteria also consumed oligosaccharides present in the hemicellulosic hydrolysates, as shown in Figure 4. *Lactobacillus casei* utilized the lowest amount and variety of oligosaccharides (Figure 4A), exhibiting preference towards xylooligosaccharides (DP = 2–4). In SG-HOS, utilization of xylobiose and xylotriose between 48 and 72 h of fermentation (Supplementary Figure S2) induced the second log phase in *L. casei*, thereby attaining higher cell counts. At approximately 60 h of growth, *L. casei* also hydrolyzed mannobiose and mannotriose in SP-HOS, leading to an increase in mannose concentration in the media (Supplementary Figure S2); however, since mannose was not a preferred carbon source, *L. casei*’s growth rate did not reach the potential maxima (Figure 2A). Xylobiose and xylotriose in HP-HOS were not metabolized until after 72 h by *L. casei*, and hence was not sufficient to promote its proliferation.

*Bacteroides fragilis* fermentation led to the reduction in concentrations of xylo-, manno-, and arabinooligosaccharides (DP = 2–4) measured in the HOS media preparations (Figure 4B). However, the corresponding growth rates in HP- and SG-HOS were lacking. This could be because, while *B. fragilis* has the transport mechanism, necessary enzymes (mannobiose 2-epimerase, mannosylglucose phosphorylase) and regulatory system to metabolize mannobiose (Kawaguchi et al., 2014), it may not have the metabolic or regulatory pathways to assimilate xylo- or arabinooligosaccharides (La Rosa et al., 2019). Hence, *B. fragilis* proliferated markedly in SP-HOS, which contained the highest concentrations of mannooligosaccharides when compared to HP and SG-HOS (Table 2).

Similarly, in *B. bifidum*, despite the reduction in concentrations of several available oligosaccharides in all HOS preparations (Figure 4C), only HP-HOS promoted the highest growth rates. Previous research has shown that several *Bifidobacterium* strains possessed the putative genes for expressing extracellular β-endoxylanase and β-xylosidase as well as α-arabinofuranosidase, conducive to hydrolyze complex arabinoxylan backbones (Rivière et al., 2014). Hence, the observed reduction in xylo- and arabino-oligosaccharide concentration in *B. bifidum* spent media may be attributed to their depolymerization into xylose and arabinose, as facilitated by the extracellular enzymes. Accordingly, the fermentation media at 120 h displayed an increase in xylose concentration by 1 and 35% in HP-HOS and SG-HOS, respectively (Supplementary Figure S4). Fermented SP-HOS media, after 120 h, also showed an increase in arabinose, glucose, and mannose concentrations by 9, 13, and 14%, respectively. This illustrates that *B. bifidum* may also have genes encoding extracellular β-mannosidases and other glucomannan degrading enzymes, although additional work is needed to substantiate this hypothesis. Overall, it could be concluded that *B. bifidum* was more efficient in degrading and utilizing complex HP-HOS, since only 1 and 2% of excess xylose and galactose, respectively, were detected in the spent media at 120 h (Supplementary Figure S4). On the other hand, *B. bifidum* exhibited prolonged lag phase in SG- and SP-HOS preparations and hence, the subsequent depolymerization and utilization of complex HOS sugars was delayed leading to comparatively lower cell counts.

It is of note that the higher-order oligosaccharides (DP > 4), which were not depleted in the fermentation media, could still impart beneficial functions when utilized as prebiotic ingredients. Studies have shown that large molecular weight oligosaccharides could improve bowel movement, gut barrier functions, anti-pathogenic activity as well as enhance the immune response in mammalian model systems (Pourabedin and Zhao, 2015; Pham et al., 2018). Alternatively, the hot water extraction conditions could be optimized to directly yield lower order HOS (DP = 2–4; Jang et al., 2021) or an additional enzymatic hydrolysis step, using endo-β-xylanase and endo-β-mannanase, could be included in order to convert the HOS into lower molecular weight (DP = 2, 3) oligosaccharides (Huang et al., 2019). Overall, the HOS substrates demonstrated in this study have immense potential for further clinical studies and exploration of bacterial assimilation mechanisms.

## Conclusion

From this study, we can conclude that, hemicellulosic hydrolysates isolated using industrially relevant hot water extraction process from dedicated bioenergy crops such as switchgrass, and hybrid poplar and from southern pine could serve as prebiotic substrates. Switchgrass HOS induced a diauxic growth pattern in *L. casei* and also resulted in the highest cell count amidst all tested probiotic bacteria and HOS substrates. In the case of hybrid poplar HOS, the initial availability of galactose led to a shorter lag phase in *B. bifidum*, whose growth rate was then sustained *via* the consumption of extracellularly depolymerized arabino- and xylooligosaccharides. This suggests that the initial establishment of probiotic bacteria and subsequent sustenance through oligosaccharide assimilation is essential to promote probiotic activity. We also observed that *B. fragilis* proliferated in southern pine HOS, exhibiting the capability to assimilate mannooligosaccharides. Although some of the underlying transport and metabolization mechanisms have been elucidated for *Lactobacillus*, *Bifidobacteria*, and *Bacteroides* species with respect to xylooligosaccharides consumption, we propose to further investigate the gene expressions specifically promoting the utilization of SG-, HP-, and SP-HOS in our future work. Overall, this study provides a promising outlook for developing high-value prebiotics from biorefinery-relevant feedstocks.

## Data Availability Statement

The original contributions presented in the study are included in the article/Supplementary Material, further inquiries can be directed to the corresponding authors.

## Author Contributions

KR conducted the experiments in conjunction with co-authors KK and JC. KR and KK also did the data analysis. NL led the conception of this study and was instrumental for the study design along with DD’S, KR, and DC. KR in conjunction with NL, DD’S, TE, and DC prepared and edited this manuscript. All authors contributed to the article and approved the submitted version.

### Conflict of Interest

The authors declare that the research was conducted in the absence of any commercial or financial relationships that could be construed as a potential conflict of interest.

## References

[ref1] Abdel-Rahman M. A. Hassan S. E.-D. Fouda A. Radwan A. A. Barghoth M. G. Desouky S. G. (2021). Evaluating the effect of lignocellulose-derived microbial inhibitors on the growth and lactic acid production by *Bacillus coagulans* Azu-10. Fermentation 7:17. 10.3390/fermentation7010017

[ref2] Ahuja K. Deb S. (2017). "Prebiotics market size by ingredient, by application, dietary supplements, industry analysis report, regional outlook, application potential, price trends, competitive market share & forecast, 2017–2024", in Food, Nutrition and Animal Feed. Selbyville, DE.

[ref3] Baral N. R. Davis R. Bradley T. H. (2019). Supply and value chain analysis of mixed biomass feedstock supply system for lignocellulosic sugar production. Biofuels Bioprod. Biorefin. 13, 635–659. 10.1002/bbb.1975

[ref4] Bozell J. J. Black S. K. Myers M. Cahill D. Miller W. P. Park S. (2011). Solvent fractionation of renewable woody feedstocks: organosolv generation of biorefinery process streams for the production of biobased chemicals. Biomass Bioenergy 35, 4197–4208. 10.1016/j.biombioe.2011.07.006

[ref5] Chaillou S. Pouwels P. H. Postma P. W. (1999). Transport of *D*-xylose in *Lactobacillus pentosus*, *Lactobacillus casei*, and *Lactobacillus plantarum*: evidence for a mechanism of facilitated diffusion via the phosphoenolpyruvate:mannose phosphotransferase system. J. Bacteriol. 181, 4768–4773. 10.1128/JB.181.16.4768-4773.1999, PMID: 10438743 PMC93960

[ref6] Chen M.-H. Bowman M. J. Cotta M. A. Dien B. S. Iten L. B. Whitehead T. R.. (2016). *Miscanthus* × *giganteus* xylooligosaccharides: purification and fermentation. Carbohydr. Polym. 140, 96–103. 10.1016/j.carbpol.2015.12.052, PMID: 26876832

[ref7] Chen M.-H. Rajan K. Carrier D. J. Singh V. (2015). Separation of xylose oligomers from autohydrolyzed *Miscanthus* x *giganteus* using centrifugal partition chromatography. Food Bioprod. Process. 95, 125–132. 10.1016/j.fbp.2015.04.006

[ref8] Das A. J. Das M. J. Miyaji T. Deka S. C. (2019). Growth and metabolic characterization of four lactic acid bacteria species isolated from rice beer prepared in Assam, India. Access Microbiol. 1:e000028. 10.1099/acmi.0.000028, PMID: 32974521 PMC7470291

[ref9] Davani-Davari D. Negahdaripour M. Karimzadeh I. Seifan M. Mohkam M. Masoumi S. J.. (2019). Prebiotics: definition, types, sources, mechanisms, and clinical applications. Foods 8:92. 10.3390/foods8030092, PMID: 30857316 PMC6463098

[ref11] Degnan B. A. Macfarlane G. T. (1995). Carbohydrate utilization patterns and substrate preferences in *Bacteroides thetaiotaomicron*. Anaerobe 1, 25–33. 10.1016/s1075-9964(95)80392-0, PMID: 16887504

[ref10] de Vries W. Stouthamer A. H. (1968). Fermentation of glucose, lactose, galactose, mannitol, and xylose by *Bifidobacteria*. J. Bacteriol. 96, 472–478. 10.1128/JB.96.2.472-478.1968, PMID: 5674058 PMC252320

[ref12] Edmunds C. W. Molina E. A. R. André N. Hamilton C. Park S. Fasina O.. (2018). Blended feedstocks for thermochemical conversion: biomass characterization and bio-oil production from switchgrass-pine residues blends. Front. Energy Res. 6:79. 10.3389/fenrg.2018.00079

[ref13] Gallina G. Cabeza Á. Grénman H. Biasi P. García-Serna J. Salmi T. (2018). Hemicellulose extraction by hot pressurized water pretreatment at 160°C for 10 different woods: yield and molecular weight. J. Supercrit. Fluids 133, 716–725. 10.1016/j.supflu.2017.10.001

[ref14] Geng W. Venditti R. A. Pawlak J. J. Chang H.-M. (2018). Effect of delignification on hemicellulose extraction from switchgrass, poplar, and pine and its effect on enzymatic convertibility of cellulose-rich residues. Bioresources 13, 4946–4963. 10.15376/biores.13.3.4946-4963

[ref15] Giacon T. G. Cunha G. C. D. G. Eliodório K. P. de Souza Oliveira P. Basso T. O. (2021). Homo-and heterofermentative lactobacilli are differently affected by lignocellulosic inhibitory compounds. bioRxiv 123. [Preprint]. 10.1101/2021.01.18.427060

[ref16] Gray J. A. Bentley J. W. Cooper J. A. Wall D. J. (2018). “Southern pulpwood production, 2015”. Asheville, NC: U.S. Department of Agriculture Forest Service, Southern Research Station, 1–15.

[ref17] Gubelt A. Blaschke L. Hahn T. Rupp S. Hirth T. Zibek S. (2020). Comparison of different lactobacilli regarding substrate utilization and their tolerance towards lignocellulose degradation products. Curr. Microbiol. 77, 3136–3146. 10.1007/s00284-020-02131-y, PMID: 32728792 PMC7452873

[ref18] Gwiazdowska D. Juś K. Jasnowska-Małecka J. Kluczyńska K. (2015). The impact of polyphenols on *Bifidobacterium* growth. Acta Biochim. Pol. 62, 895–901. 10.18388/abp.2015_1154, PMID: 26619254

[ref19] Hansen C. H. Frøkiær H. Christensen A. G. Bergström A. Licht T. R. Hansen A. K.. (2013). Dietary xylooligosaccharide downregulates IFN-γ and the low-grade inflammatory cytokine IL-1β systemically in mice. J. Nutr. 143, 533–540. 10.3945/jn.112.172361, PMID: 23427328

[ref20] Hill D. Sugrue I. Tobin C. Hill C. Stanton C. Ross R. P. (2018). The *Lactobacillus casei* group: history and health related applications. Front. Microbiol. 9:2107. 10.3389/fmicb.2018.02107, PMID: 30298055 PMC6160870

[ref21] Hong C. Y. Corbett D. Venditti R. Jameel H. Park S. (2019). Xylooligosaccharides as prebiotics from biomass autohydrolyzate. LWT 111, 703–710. 10.1016/j.lwt.2019.05.098

[ref22] Hsu C. K. Liao J. W. Chung Y. C. Hsieh C. P. Chan Y. C. (2004). Xylooligosaccharides and fructooligosaccharides affect the intestinal microbiota and precancerous colonic lesion development in rats. J. Nutr. 134, 1523–1528. 10.1093/jn/134.6.1523, PMID: 15173423

[ref23] Huang C. Wang X. Liang C. Jiang X. Yang G. Xu J.. (2019). A sustainable process for procuring biologically active fractions of high-purity xylooligosaccharides and water-soluble lignin from *Moso* bamboo prehydrolyzate. Biotechnol. Biofuels 12:189. 10.1186/s13068-019-1527-3, PMID: 31384296 PMC6661736

[ref24] Jang S.-K. Kim J.-H. Choi J.-H. Cho S.-M. Kim J.-C. Kim H.. (2021). Evaluation of xylooligosaccharides production for a specific degree of polymerization by liquid hot water treatment of tropical hardwood. Foods 10:463. 10.3390/foods10020463, PMID: 33672511 PMC7923788

[ref25] Karnaouri A. Matsakas L. Krikigianni E. Rova U. Christakopoulos P. (2019). Valorization of waste forest biomass toward the production of cello-oligosaccharides with potential prebiotic activity by utilizing customized enzyme cocktails. Biotechnol. Biofuels 12:285. 10.1186/s13068-019-1628-z, PMID: 31827613 PMC6902470

[ref26] Kawaguchi K. Senoura T. Ito S. Taira T. Ito H. Wasaki J.. (2014). The mannobiose-forming exo-mannanase involved in a new mannan catabolic pathway in *Bacteroides fragilis*. Arch. Microbiol. 196, 17–23. 10.1007/s00203-013-0938-y, PMID: 24217874

[ref27] Kilpeläinen P. O. Hautala S. S. Byman O. O. Tanner L. J. Korpinen R. I. Lillandt M. K.-J.. (2014). Pressurized hot water flow-through extraction system scale up from the laboratory to the pilot scale. Green Chem. 16, 3186–3194. 10.1039/C4GC00274A

[ref28] Kim Y. Hendrickson R. Mosier N. S. Ladisch M. R. (2009). “Liquid hot water pretreatment of cellulosic biomass” in Biofuels: Methods in Molecular Biology. ed. Mielenz J. (Totowa, NJ: Humana Press), 93–102. 10.1007/978-1-60761-214-8_7 19768618

[ref29] Kim S. Kim S. Cho J. Park S. Perez F. X. J. Kiniry J. R. (2020). Simulated biomass, climate change impacts, and n itrogen management to achieve switchgrass biofuel production at diverse sites in U.S. Agronomy 10:503. 10.3390/agronomy10040503

[ref30] Krogell J. Korotkova E. Eränen K. Pranovich A. Salmi T. Murzin D.. (2013). Intensification of hemicellulose hot-water extraction from spruce wood in a batch extractor—effects of wood particle size. Bioresour. Technol. 143, 212–220. 10.1016/j.biortech.2013.05.110, PMID: 23792759

[ref31] La Rosa S. L. Kachrimanidou V. Buffetto F. Pope P. B. Pudlo N. A. Martens E. C.. (2019). Wood-derived dietary fibers promote beneficial human gut microbiota. mSphere 4, e00554–e00618. 10.1128/mSphere.00554-18, PMID: 30674645 PMC6344601

[ref32] Licandro-Seraut H. Scornec H. Pédron T. Cavin J.-F. Sansonetti P. J. (2014). Functional genomics of *Lactobacillus casei* establishment in the gut. Proc. Natl. Acad. Sci. U. S. A. 111, E3101–E3109. 10.1073/pnas.1411883111, PMID: 25024222 PMC4121828

[ref33] Lira C. (2018). Autohydrolysis pretreatment of mixed lignocellulosic biomass. Doctor of Philosophy. Ontario, Canada: The University of Western Ontario, 142.

[ref34] Liu Q. Labbé N. Adhikari S. Chmely S. C. Abdoulmoumine N. (2018). Hot water extraction as a pretreatment for reducing syngas inorganics impurities—A parametric investigation on switchgrass and loblolly pine bark. Fuel 220, 177–184. 10.1016/j.fuel.2018.01.108

[ref35] Lolou V. Panayiotidis M. I. (2019). Functional role of probiotics and prebiotics on skin health and disease. Fermentation 5:41. 10.3390/fermentation5020041

[ref36] Moura P. Barata R. Carvalheiro F. Gírio F. Loureiro-Dias M. C. Esteves M. P. (2007). In vitro fermentation of xylo-oligosaccharides from corn cobs autohydrolysis by *Bifidobacterium* and *Lactobacillus* strains. LWT Food Sci. Technol. 40, 963–972. 10.1016/j.lwt.2006.07.013

[ref37] Ng S. Y. Chia L. W. Padam B. S. Chye F. Y. (2014). Effect of selected oligosaccharides on the viability and fermentation kinetics of *Lactobacillus acidophilus* and *Lactobacillus casei* in cultured milk. J. Pharm. Nutr. Sci. 4, 92–99. 10.6000/1927-5951.2014.04.02.4

[ref38] Pacheco-Ordaz R. Wall-Medrano A. Goni M. G. Ramos-Clamont-Montfort G. AyalaZavala J. F. Gonzalez-Aguilar G. A. (2017). Effect of phenolic compounds on the growth of selected probiotic and pathogenic bacteria. Lett. Appl. Microbiol. 66, 25–31. 10.1111/lam.12814, PMID: 29063625

[ref39] Patipong T. Lotrakul P. Padungros P. Punnapayak H. Bankeeree W. Prasongsuk S. (2019). Enzymatic hydrolysis of tropical weed xylans using xylanase from *Aureobasidium melanogenum* PBUAP46 for xylooligosaccharide production. 3 Biotech 9:56. 10.1007/s13205-019-1586-y, PMID: 30729080 PMC6349264

[ref40] Phaiboonsilpa N. Chysirichote T. Champreda V. Laosiripojana N. (2020). Fermentation of xylose, arabinose, glucose, their mixtures and sugarcane bagasse hydrolyzate by yeast *Pichia stipitis* for ethanol production. Energy Rep. 6, 710–713. 10.1016/j.egyr.2019.11.142

[ref41] Pham V. T. Seifert N. Richard N. Raederstorff D. Steinert R. Prudence K.. (2018). The effects of fermentation products of prebiotic fibres on gut barrier and immune functions in vitro. PeerJ 6:e5288. 10.7717/peerj.5288, PMID: 30128177 PMC6089210

[ref42] Poeker S. A. Geirnaert A. Berchtold L. Greppi A. Krych L. Steinert R. E.. (2018). Understanding the prebiotic potential of different dietary fibers using an in vitro continuous adult fermentation model (PolyFermS). Sci. Rep. 8:4318. 10.1038/s41598-018-22438-y, PMID: 29531228 PMC5847601

[ref43] Pourabedin M. Zhao X. (2015). Prebiotics and gut microbiota in chickens. FEMS Microbiol. Lett. 362:122. 10.1093/femsle/fnv122, PMID: 26208530

[ref44] Rajan K. Carrier D. J. (2016). Insights into exo-cellulase inhibition by the hot water hydrolyzates of rice straw. ACS Sustain. Chem. Eng. 4, 3627–3633. 10.1021/acssuschemeng.5b01778

[ref45] Ratnadewi A. A. I. Zain M. H. A. Kusuma A. A. N. N. Handayani W. Nugraha A. S. Siswoyo T. A. (2020). *Lactobacillus casei* fermentation towards xylooligosaccharide (XOS) obtained from coffee peel enzymatic hydrolysate. Biocatal. Agric. Biotechnol. 23:101446. 10.1016/j.bcab.2019.101446

[ref46] Rivière A. Moens F. Selak M. Maes D. Weckx S. De Vuyst L. (2014). The ability of *Bifidobacteria* to degrade arabinoxylan oligosaccharide constituents and derived oligosaccharides is strain dependent. Appl. Environ. Microbiol. 80, 204–217. 10.1128/AEM.02853-13, PMID: 24141124 PMC3911024

[ref47] Rusanen A. Lappalainen K. Kärkkäinen J. Tuuttila T. Mikola M. Lassi U. (2019). Selective hemicellulose hydrolysis of scots pine sawdust. Biomass Convers. Biorefin. 9, 283–291. 10.1007/s13399-018-0357-z

[ref48] Sims I. M. Tannock G. W. (2020). Galacto- and fructo-oligosaccharides utilized for growth by cocultures of bifidobacterial species characteristic of the infant gut. Appl. Environ. Microbiol. 86, e00214–e00220. 10.1128/AEM.00214-20, PMID: 32220841 PMC7237773

[ref49] Sluiter A. Hames B. Ruiz R. Scarlata C. Sluiter J. Templeton D. (2008). “Determination of sugars, byproducts, and degradation products in liquid fraction process samples” in Laboratory Analytical Procedure. (Golden, Colorado: National Renewable Energy Laboratory), 1–14.

[ref50] Sluiter J. B. Ruiz R. O. Scarlata C. J. Sluiter A. D. Templeton D. W. (2010). Compositional analysis of lignocellulosic feedstocks. 1. Review and description of methods. J. Agric. Food Chem. 58, 9043–9053. 10.1021/jf1008023, PMID: 20669951 PMC2923870

[ref51] Spiridon I. Popa V. I. (2008). “Hemicelluloses: major sources, properties and applications” in Monomers, Polymers and Composites From Renewable Resources. eds. Belgacem M. N. Gandini A. (Amsterdam, Netherlands: Elsevier), 289–304.

[ref52] Takkellapati S. Li T. Gonzalez M. A. (2018). An overview of biorefinery derived platform chemicals from a cellulose and hemicellulose biorefinery. Clean Technol. Environ. Policy 20, 1615–1630. 10.1007/s10098-018-1568-5, PMID: 30319323 PMC6178844

[ref53] Thiennimitr P. Yasom S. Tunapong W. Chunchai T. Wanchai K. Pongchaidecha A.. (2018). *Lactobacillus paracasei* HII01, xylooligosaccharides, and synbiotics reduce gut disturbance in obese rats. Nutrition 54, 40–47. 10.1016/j.nut.2018.03.005, PMID: 29705500

[ref54] Townsend P. A. Haider N. Boby L. Heavey J. Miller T. A. Volk T. A. (2018). “A roadmap for polar and willow to provide environmental services to build the bioeconomy” in Environmental Applications. eds. Hart N. Isebrands, J. Johnston C. Licht L. Shell M. Simmons B.. (Pullman, WA: Washington State University), 1–36.

[ref55] Turroni F. Milani C. Duranti S. Lugli G. A. Bernasconi S. Margolles A.. (2020). The infant gut microbiome as a microbial organ influencing host well-being. Ital. J. Pediatr. 46:16. 10.1186/s13052-020-0781-0, PMID: 32024556 PMC7003403

[ref56] Turroni F. Strati F. Foroni E. Serafini F. Duranti S. Van Sinderen D.. (2012). Analysis of predicted carbohydrate transport systems encoded by B*ifidobacterium bifidum* PRL2010. Appl. Environ. Microbiol. 78, 5002–5012. 10.1128/AEM.00629-12, PMID: 22562993 PMC3416360

[ref57] Volk T. A. Berguson B. Daly C. Halbleib M. Miller R. Rials T.. (2018). Poplar and shrub willow energy crops in the United States: field trial results from the multiyear regional feedstock partnership and yield potential maps based on the PRISM-ELM model. GCB Bioenergy 10, 735–751. 10.1111/gcbb.12498

[ref58] Wang J. Boy R. Nguyen N. A. Keum J. K. Cullen D. A. Chen J.. (2017). Controlled assembly of lignocellulosic biomass components and properties of reformed materials. ACS Sustain. Chem. Eng. 5, 8044–8052. 10.1021/acssuschemeng.7b01639

[ref59] Wells J. M. Drielak E. Surendra K. C. Khanal S. K. (2020). Hot water pretreatment of lignocellulosic biomass: modeling the effects of temperature, enzyme and biomass loadings on sugar yield. Bioresour. Technol. 300:122593. 10.1016/j.biortech.2019.122593, PMID: 31881517

[ref60] Yadav M. Paritosh K. Chawade A. Pareek N. Vivekanand V. (2018). Genetic engineering of energy crops to reduce recalcitrance and enhance biomass digestibility. Agriculture 8:76. 10.3390/agriculture8060076

[ref61] Yan L. Ma R. Li L. Fu J. (2016). Hot water pretreatment of lignocellulosic biomass: an effective and environmentally friendly approach to enhance biofuel production. Chem. Eng. Techol. 39, 1759–1770. 10.1002/ceat.201600394

[ref62] Yang J. Summanen P. H. Henning S. M. Hsu M. Lam H. Huang J.. (2015). Xylooligosaccharide supplementation alters gut bacteria in both healthy and prediabetic adults: a pilot study. Front. Physiol. 6:216. 10.3389/fphys.2015.00216, PMID: 26300782 PMC4528259

