# Supplementary material for: Production and Characterization of High Value Prebiotics From Biorefinery-Relevant Feedstocks
Source: Front Microbiol. 2021 Apr 29;12:675314. doi: 10.3389/fmicb.2021.675314 (PMC8116503; doi:10.3389/fmicb.2021.675314)
Supplement: Supplementary file 1 [file Data_Sheet_1.DOCX]

Supplemental information

Production and Characterization of High Value Prebiotics from Biorefinery-relevant Feedstocks

*Kalavathy Rajan^1‡^, Doris D’Souza^2^, Keonhee Kim^1^, Joseph Moon Choi^2^, Thomas Elder^3^, Danielle Julie Carrier^4^, Nicole Labbé^1, 5*^*

^1^Center for Renewable Carbon, The University of Tennessee Institute of Agriculture, Knoxville, Tennessee 37996, USA.

^2^Department of Food Science, The University of Tennessee Institute of Agriculture, Knoxville, Tennessee 37996, USA.

^3^USDA-Forest Service, Southern Research Station, Auburn, AL 36849, USA.

^4^Department of Biosystems Engineering & Soil Science, The University of Tennessee Institute of Agriculture, Knoxville, Tennessee 37996, USA.

^5^Department of Forestry, Wildlife and Fisheries, The University of Tennessee Institute of Agriculture, Knoxville, Tennessee 37996, USA.

**^* ‡^Corresponding authors**

**TABLE OF CONTENTS Page #**

Figure S1. Flow cytometry cell counts and optical density calibration S1

Figure S2. Chemical composition of media during *L. casei* fermentation S2

Figure S3. Chemical composition of media during *B. fragilis* fermentation S3

Figure S4. Chemical composition of media during *B. bifidum* fermentation S4

Table S1. Concentration of degradation products in hemicellulosic hydrolysates S5


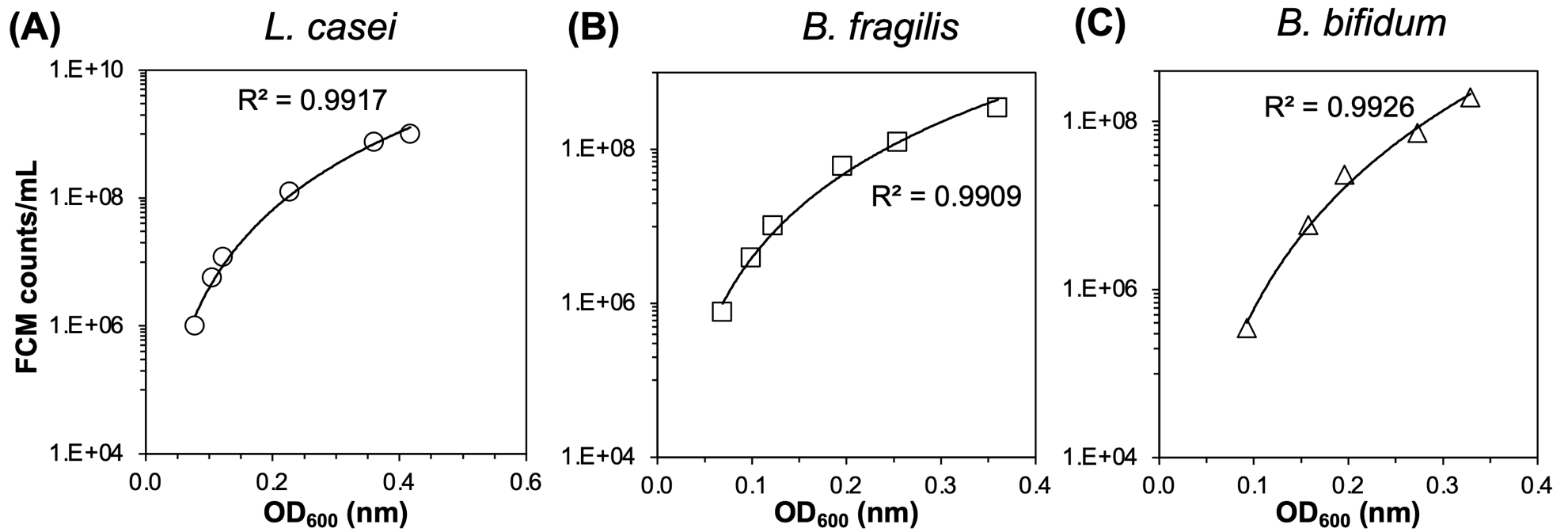


**Figure S1.** Calibration of bacterial cell densities, measured using a UV-Vis spectrophotometer at 600 nm, based on the flow cytometer (FCM) counts for (A) *Lactobacillus casei*, (B) *Bacteroides fragilis* and (C) *Bifidobacterium bifidum*. A power function was used for curve fitting. Bacteria pelleted from their corresponding growth media were resuspended in 5% tryptic soy broth (TSB) and diluted to different concentrations such that they were conducive for FCM measurements. TSB blanks and microsphere blank (i.e., bacteria without internal standard) were used to optimize the instrument method.

Briefly, the Invitrogen™ bacteria counting kit (catalog #B7277) purchased from Thermo Fisher Scientific (Rockwood, TN) was used for flow cytometry measurements. Preliminary experiments were conducted in order to optimize the following instrument parameters for every bacterial species; (a) forward scatter, (b) side scatter and (c) threshold scatter by running TSB and microsphere blanks; (d) minimum and maximum dilutions for the resuspended bacterial cells. Fresh bacterial cultures in mid-log phase were used for all experiments. To 1 mL of diluted media containing the test bacterium, 5 μl of the SYTO^®^ bacterial stain was added and incubated for 5 min at 37 °C. Then 20 μl of freshly resuspended microspheres was added, vortexed well, and immediately subjected to flow cytometry. Microspheres function as internal standard, where one sphere equals to one bacterium per 10^-6^ mL. Total cells (viable plus non-viable cells) were calculated based on the ratio of bacteria count over the microsphere count.


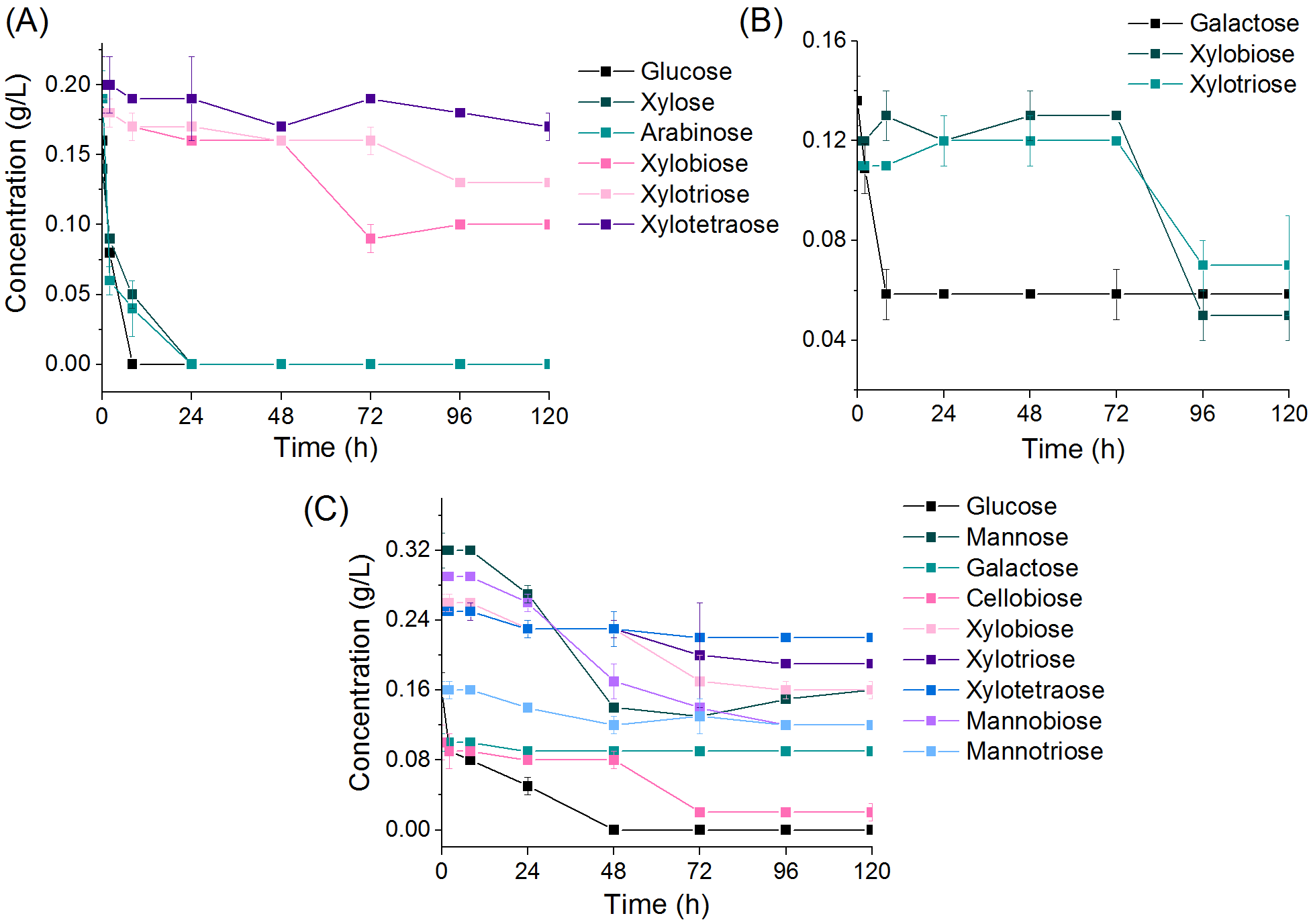


**Figure S2.** Concentration of mono- and oligosaccharides in the hemicellulosic hydrolysates (4 g/L) as a function of time during fermentation by *Lactobacillus casei*. The hemicellulosic hydrolysates were prepared from (A) Switchgrass, (B) Hybrid poplar and (C) Southern pine using hot water extraction at 160 °C for 60 min.


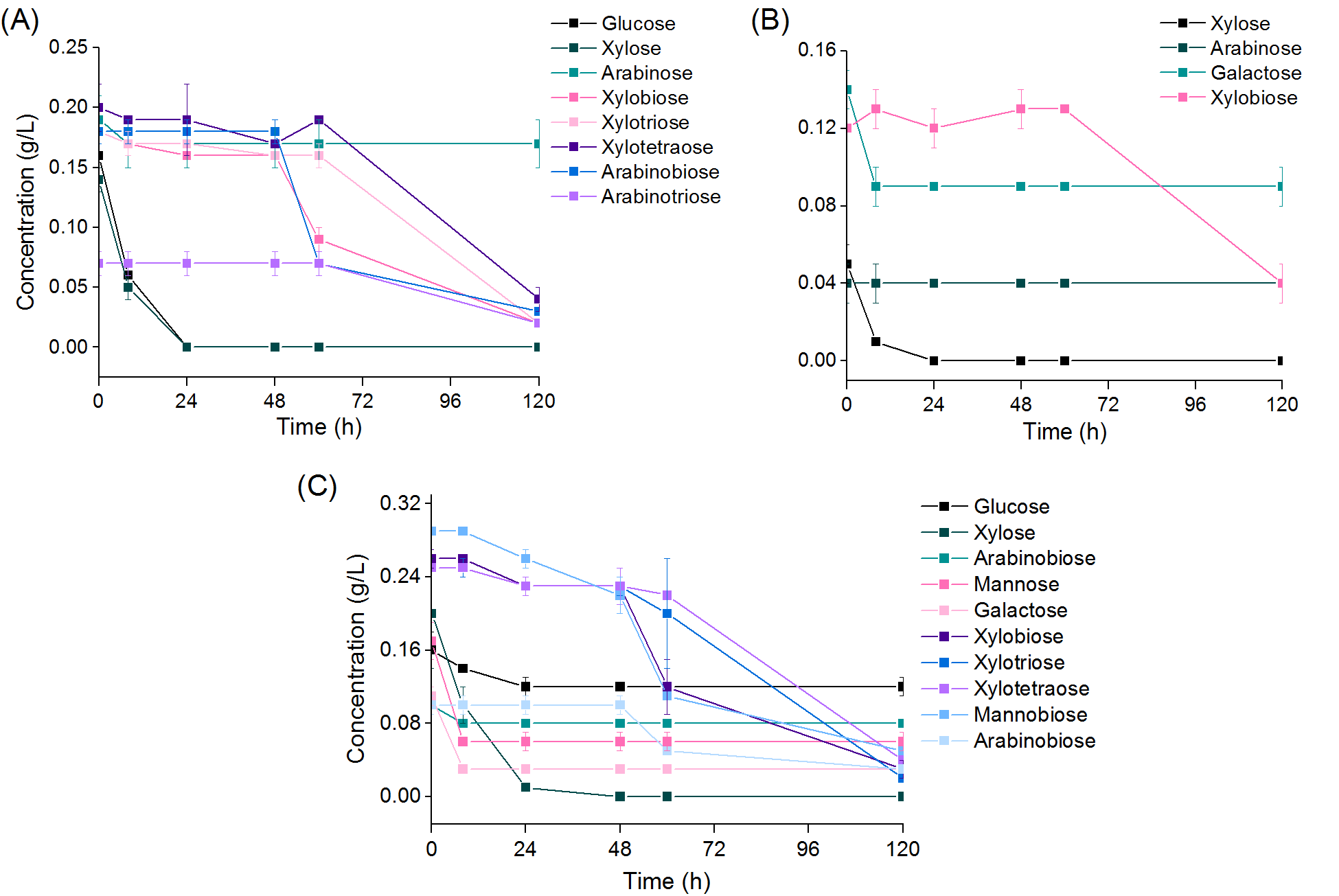


**Figure S3.** Concentration of mono- and oligosaccharides in the hemicellulosic hydrolysates (4 g/L) as a function of time during fermentation by *Bacteroides fragilis*. The hemicellulosic hydrolysates were prepared from (A) Switchgrass, (B) Hybrid poplar and (C) Southern pine using hot water extraction at 160 °C for 60 min.


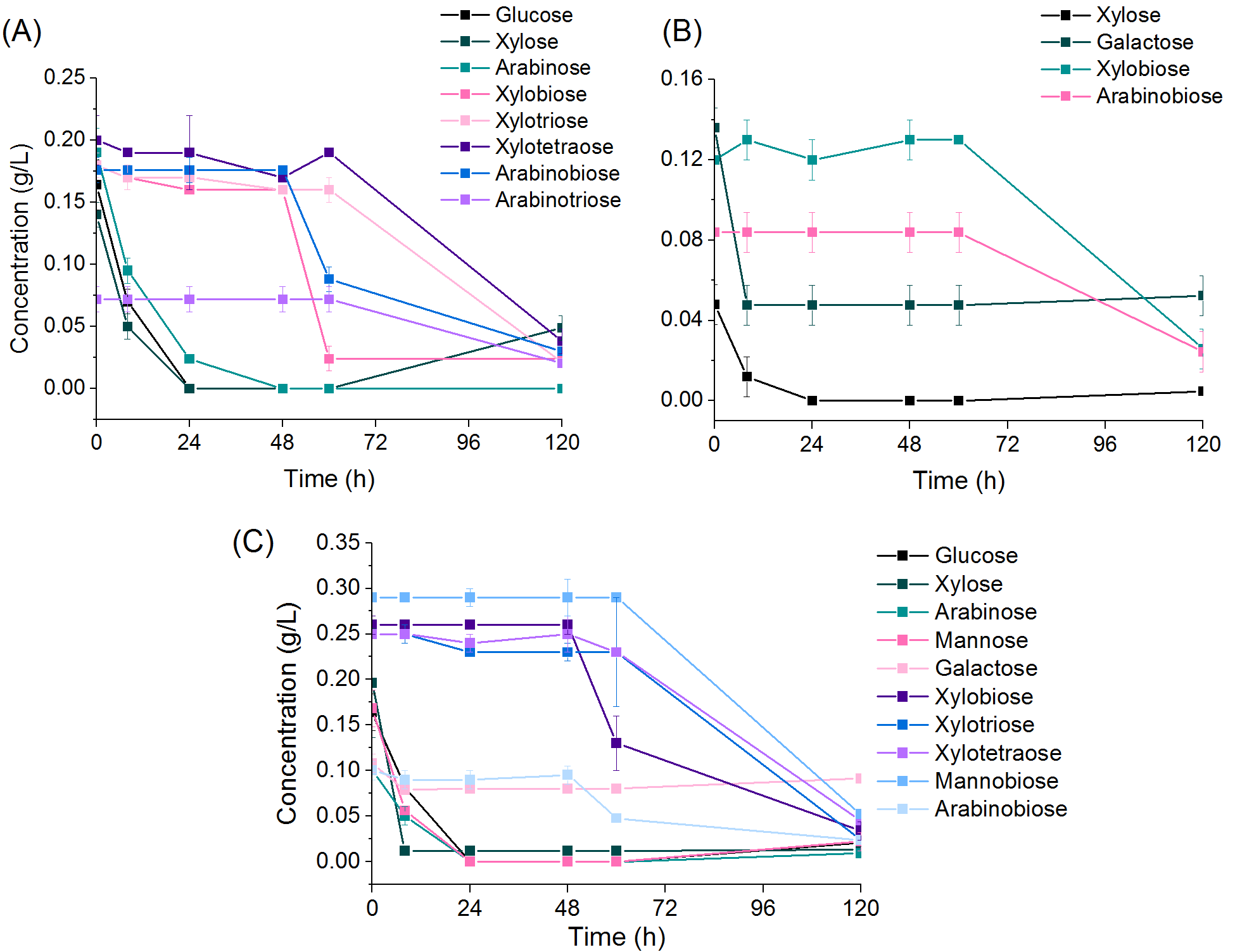


**Figure S4.** Concentration of mono- and oligosaccharides in the hemicellulosic hydrolysates (4 g/L) as a function of time during fermentation by *Bifidobacterium bifidum*. The hemicellulosic hydrolysates were prepared from (A) Switchgrass, (B) Hybrid poplar and (C) Southern pine using hot water extraction at 160 °C for 60 min.

| **Table S1.** Concentration of degradation products in hemicellulosic oligosaccharides (HOS) | | | |
| --- | --- | --- | --- |
| **Compound (g/L)** | **SG-HOS** | **HP-HOS** | **SP-HOS** |
| Acetic acid | 0.7 ± 0.1 | n.d. | 13.4 ± 1.4 |
| Formic acid | 5.3 ± 0.6 | n.d. | 9.7 ± 0.8 |
| Furfural | 2.0 ± 0.0 | n.d. | 2.3 ± 0.1 |
| 5-Hydroxymethylfurfural | 0.8 ± 0.2 | n.d. | 2.2 ± 0.2 |
| Total phenolics (GAE)* | 10.3 ± 1.0 | 7.4 ± 0.1 | 4.0 ± 0.1 |
| Average (± standard deviation) concentrations for all HOS are provided for *N* = 3; SG – Switchgrass, HP – Hybrid poplar, SP – Southern pine.  *GAE - Gallic acid equivalent. | | | |
